# The cerebral metabolic mechanism of group computer magnanimous therapy based on magnetic resonance spectroscopy: effects on improving magnanimous-enterprising levels of lung cancer patients

**DOI:** 10.3389/fpsyt.2024.1397375

**Published:** 2024-12-10

**Authors:** Qianyu Liu, Qihui Ma, Qingfeng Sun, Xuewei Huang

**Affiliations:** ^1^ Psychological Clinic, Guangxi Chest Hospital, Liuzhou, Guangxi, China; ^2^ Medical Department, Huizhou Central People’s Hospital, Huizhou, Guangdong, China; ^3^ Department of Pulmonary Medicine, Guangxi Chest Hospital, Liuzhou, Guangxi, China; ^4^ Department of Psychiatry & Medical Psychology, The First Affiliated Hospital, College of Clinical Medicine, Guangdong Pharmaceutical University, Guangzhou, Guangdong, China

**Keywords:** group computer magnanimous therapy, magnanimous-enterprising level, brain metabolism, lung cancer, psychosomatic effects

## Abstract

**Introduction:**

This study aims to evaluate the effects of group computer magnanimous therapy (GCMT) on magnanimous-enterprising levels and brain metabolic changes in patients with advanced lung cancer.

**Methods:**

In this multicenter, randomized controlled trial, 47 participants diagnosed with advanced stage (III or IV) lung cancer were randomly assigned to either the GCMT group (GCMTG, *n* = 31) or the control group (CTRLG, *n* = 16). The GCMTG received routine oncotherapy and care along with eight sessions of GCMT over 2 weeks, while the CTRLG received only oncotherapy and routine care. Psychological and brain metabolic changes were assessed using the Enterprising and Magnanimous Questionnaire (EMQ) and proton magnetic resonance spectroscopy (^1^H-MRS).

**Results:**

After 2 weeks, the GCMTG showed significant improvements in the EMQ “total score” and “enterprising” dimensions compared to baseline (*p* < 0.05), while the CTRLG showed no significant changes. Significant increases in NAA/Cr levels were observed in the right amygdala, and significant decreases in mI/Cr levels were observed in the right cingulate gyrus in the GCMTG. Pearson correlation analysis indicated that changes in Cho/Cr levels in the left amygdala and Glx/Cr levels in the left hippocampus were significantly correlated with improvements in the enterprising dimension.

**Conclusions:**

GCMT significantly enhanced enterprising attitudes and induced beneficial changes in brain metabolites among patients with advanced lung cancer. Further research with larger sample sizes is warranted to confirm these results and explore the long-term effects of GCMT.

**Clinical trial registration:**

https://www.chictr.org.cn/showproj.html?proj=129557, identifier ChiCTR2100053015.

## Introduction

Lung cancer is a malignant tumor with the highest mortality throughout the world (1, 2). According to the World Health Organization (WHO), lung cancer has surpassed gastric cancer as the main cause of cancer death, accounting for 27.3% of all cancer deaths in China (3). The onset of lung cancer is hidden, and the early symptoms are easy to be ignored (4–6). When diagnosed, it is often in the advanced stage of the disease, which brings huge psychological and physiological changes to patients (7). This not only increases the complexity of treatment but also profoundly impacts the psychological health of patients.

Research indicates that patients with advanced lung cancer frequently face severe psychological issues, including depression, anxiety, despair, and social withdrawal (8, 9). These psychological problems may lead to decreased treatment compliance, significantly impair quality of life, and even affect survival rates (10). For instance, a study by Smith et al. found a significant correlation between depressive symptoms and high mortality rates among lung cancer patients (11). Additionally, psychological issues may exacerbate physical symptoms such as pain and fatigue, creating a vicious cycle.

Magnetic resonance spectroscopy (MRS) is a non-invasive examination method used to analyze the neurobiochemical metabolism and pathophysiology of human organs and tissues. ¹H-MRS is the most widely used in studying changes in biochemistry and metabolites in the brain (12). In recent years, a growing number of researchers (13–16) have conducted MRS research on psychiatric mental illness. Huang BJ and Yang Y conducted a 2-month group cognitive behavior therapy on patients with Internet addiction. The results showed that after the intervention, the Cho/Cr ratio of the medial prefrontal lobe was significantly higher than that before treatment, and the behavior of Internet addiction was improved (17, 18). The study of Streeter CC et al. (19) showed that after a 3-month period of exercise therapy, the level of gamma-aminobutyric acid (GABA) in the thalamus increased significantly, while the level of anxiety was lower than before. It has been shown that the concentration of Glx in the right caudate nucleus decreased and the compulsive behavior gradually decreased in obsessive–compulsive disorder patients after 4 months of behavioral therapy (20). However, few empirical studies exist on cerebral metabolic mechanisms in advanced lung cancer patients with psychotherapy. Therefore, it is urgent to collect additional evidence on brain metabolic transformations in patients with advanced lung cancer.

Magnanimous therapy (MT) is a brand-new psychotherapy method proposed by Huang X (21), based on over 20 years of clinical treatment experience of research groups and research on cancer psychotherapy at home and abroad. It combines the cultural and psychological habits of Chinese people and integrates the essence of Chinese civilization, Zen, and Taoism. The purpose of treatment is to present the magnanimous and open-minded, enterprising and positive, and optimistic attitude toward life to individuals in a simple, interesting, and vivid way. In a 2021 study, we assessed the psychological status of lung cancer patients using the Psychosomatic Status Scale for Cancer Patients (PSSCP) and the Hospital Anxiety Depression Scale (HADS); additionally, we evaluated immune function by measuring plasma levels of immunoglobulins IgA, IgG, and IgM, as well as natural killer (NK) cell activity; our findings indicate that MT contributed to improvements in depression, anxiety, psychosomatic status, and immune function in patients with advanced lung cancer (22). Our early studies (23–28) found that patients with breast cancer and lung cancer had positive improvements in physical symptoms, behavior patterns, social functions, and emotional status, through therapeutic intervention of MT. GCMT was an improved psychotherapy based on the technology and theory of MT, which combines MT, group psychotherapy, and game psychotherapy, so that more patients can participate in the treatment under the guidance of therapists.

GCMT has been applied to different kinds of diseases. However, the therapeutic role and mechanism of GCMT in lung cancer are largely unknown. The effects of psychotherapy on the magnanimous-enterprising level and cerebral metabolic mechanism of advanced lung cancer patients have not yet been tested. In order to fill the current research gap, this study aims to 1) evaluate the magnanimous-enterprising levels and brain metabolic levels of GCMT in advanced lung cancer patients, 2) reveal the relationship between the effects of GCMT on magnanimous-enterprising and brain metabolites, and 3) explore the possible mechanism of GCMT in lung cancer patients (2). To our knowledge, this study is the first of its kind to explore the cerebral metabolic mechanism of GCMT in patients with lung cancer.

## Methods

### Study design

This two‐arm, parallel, blocked, multicenter, randomized controlled trial (RCT) study was approved by the Clinical Medical Ethics Committee [Approval No. 2013 Clinical Medicine (06)] and performed in line with the principles of the Declaration of Helsinki. This study aimed to investigate the effects of GCMT on magnanimous-enterprising and brain metabolite levels in patients with advanced lung cancer. Participants were randomly assigned to the group computer magnanimous therapy group (GCMTG) or the control group (CTRLG). The control group only received routine oncotherapy and nursing, while the GCMT group received eight times GCMT on this basis. Patients were assessed at baseline and 2 weeks later using the Enterprising and Magnanimous Questionnaire (EMQ) and MRS brain metabolic indexes [the peak heights of N-acetylaspartate (NAA), choline (Cho), myo-inositol (mI), glutamate, and glutamine complex (Glx) in the bilateral anterior cingulate gyrus, hippocampus, and amygdala were collected by ^1^H-MRS, and the spectrum was analyzed relatively quantitatively with reference to creatine (CR) peak, and the values of NAA/Cr, Cho/Cr, mI/Cr, and Glx/Cr were recorded]. The relationship between the changes in magnanimous-enterprising levels and brain biochemical metabolism was analyzed to explore the possible mechanism.

### Participants

**Figure 1:**
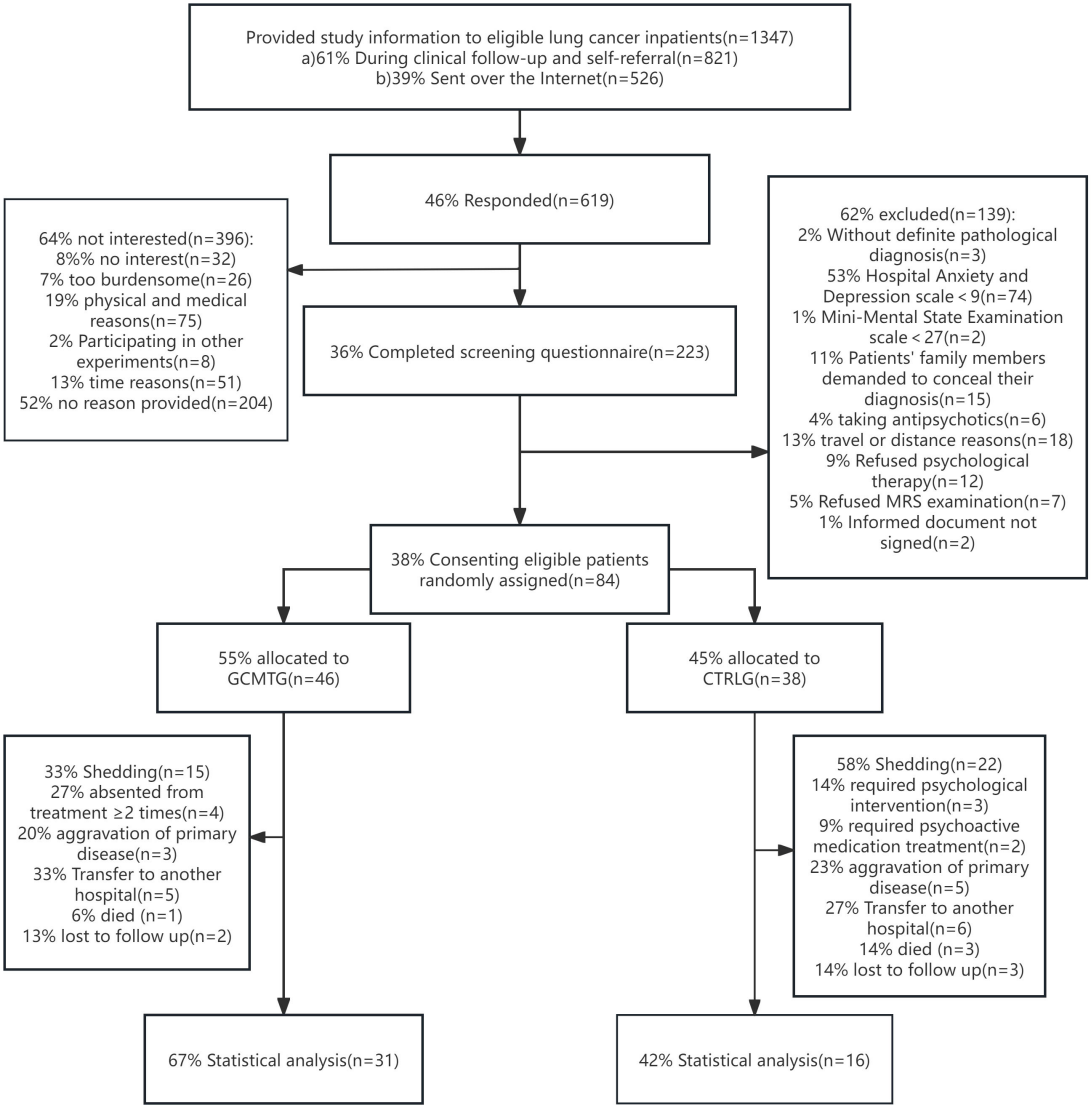
The CONSORT flow chart shows the recruitment and enrollment of 47 participants for final statistical analysis.

Consecutive eligible patients were recruited at the oncology inpatient department of three teaching hospitals in Guangdong, China, from 2014 to 2021. Relevant study information was provided through prospective contact by doctors or nurses during routine visits or through online recruitment (WeChat, Moments, Public Accounts). For interested patients, after an interview by the researchers, an eligibility assessment was conducted through paper-based screening questionnaires; eligible patients signed a written informed consent. A cohort comprising 84 individuals diagnosed with advanced stage (III or IV) lung carcinoma was initially enrolled in the study. Due to dropouts during the course of therapy and subsequent follow-up, the dataset was reduced to 47 subjects for the final statistical analysis (**Figure 1**).

The inclusion criteria were as follows: 1) age range: 25–85 years old, primary education or above; 2) have a definite pathological diagnosis; 3) Hospital Anxiety and Depression (HAD) scale ≥9, Mini-Mental State Examination (MMSE) scale ≥27; 4) patients with stable condition after clinical oncology therapy; 5) no obvious intellectual disability or psychiatric disorders; and 6) patients voluntarily participated and signed informed documents.

The exclusion criteria were 1) without a definite pathological diagnosis, 2) patients’ family members demanded to conceal their diagnosis, 3) with cognitive impairment (MMSE < 27), 4) taking antipsychotics or receiving other psychotherapy, 5) a history of psychoactive drug abuse, 6) pregnant or lactating women, and 7) other reasons for being unable to accept psychological intervention.

The shedding criteria were as follows: 1) absent from treatment on two or more occasions, 2) could not continue to receive psychotherapy because of the aggravation of the primary disease and unstable vital signs, 3) with cognitive impairment due to brain metastasis during the experiment, and 4) participants in the control group who needed other psychological intervention or psychotropic drug therapy due to their condition.

### Randomization and masking

This blocked RCT involved participants who, subsequent to baseline assessments, were stratified by gender (male or female), educational level, cancer diagnosis (pathological classification and clinical stage), and therapeutic approaches. Eligible subjects were randomly assigned in a 1:1 ratio to either the GCMTG or the CTRLG using block randomization with varying block sizes of two to four participants. The randomization was facilitated by an online program developed in-house by the hospital’s Information Technology Department, which ensured immediate allocation. Participants allocated to the GCMTG received an invitation to book a session with a psychologist. Neither the participants nor the research staff were blinded to the intervention status, which is common in psychological intervention trials and is understood as part of the intervention, akin to clinical practice. The statistician entrusted with data analysis remained blinded to the intervention allocation.

### Intervention

Before the intervention, the patients in the experimental group were randomly divided into treatment groups of three to four people. Through careful observation and patient communication, the therapist initially understood the psychosomatic state of the group members, introduced the treatment-related situation to the patients, and initially established a treatment relationship with the group members. GCMT was implemented with computer games as the carrier (play therapy games, the games focused on inspiring a positive interaction effect in a group). After each game, the therapist guided the patients to comprehend, share, and communicate; made the patients resonate and introspect; and required each of them to spend 30 min every day recalling the treatment content and apply what they have learned to their daily lives. At the beginning of the next session, the therapist led the members to review the last learning content and check the completion of the homework, so that they could share their feelings on the application. Participants in the GCMT group received an eight-time course of GCMT (4 times per week for 2 weeks). (The length of hospital stay of Chinese lung cancer patients is usually 2 weeks.) Through a 2-week continuous learning and daily consolidation, the participants could discuss, support, and encourage each other; gradually experience and understand the beliefs and essence of a magnanimous, enterprising, and optimistic attitude; and achieve a relaxing, harmonious, and peaceful state.

### Outcome measurements

Magnanimous-enterprising levels were assessed using the 23-item EMQ self-rating scale, prepared by Yang R et al. in 2014 (29). This scale consists of 2 dimensions and 23 questions; the lower the score of each dimension, the less aggressive or open-minded the patient is. On the other hand, they have corresponding magnanimous or enterprising psychological and behavioral characteristics. This scale has good reliability and validity; the test–retest coefficients for the two dimensions and the total scores were from 0.710 to 0.825. The split-half reliability for the scale was 0.861. The calibration validity of the 16PF Personality Questionnaire on the boldness factor and stability factor was 0.623 and 0.728. Given our previous report on the effects of MT on emotional outcomes (22), this study focuses on exploring the impact on magnanimous-enterprising levels, thus omitting the repeated use of the PSSCP and HADS scales.

**Figure 2:**
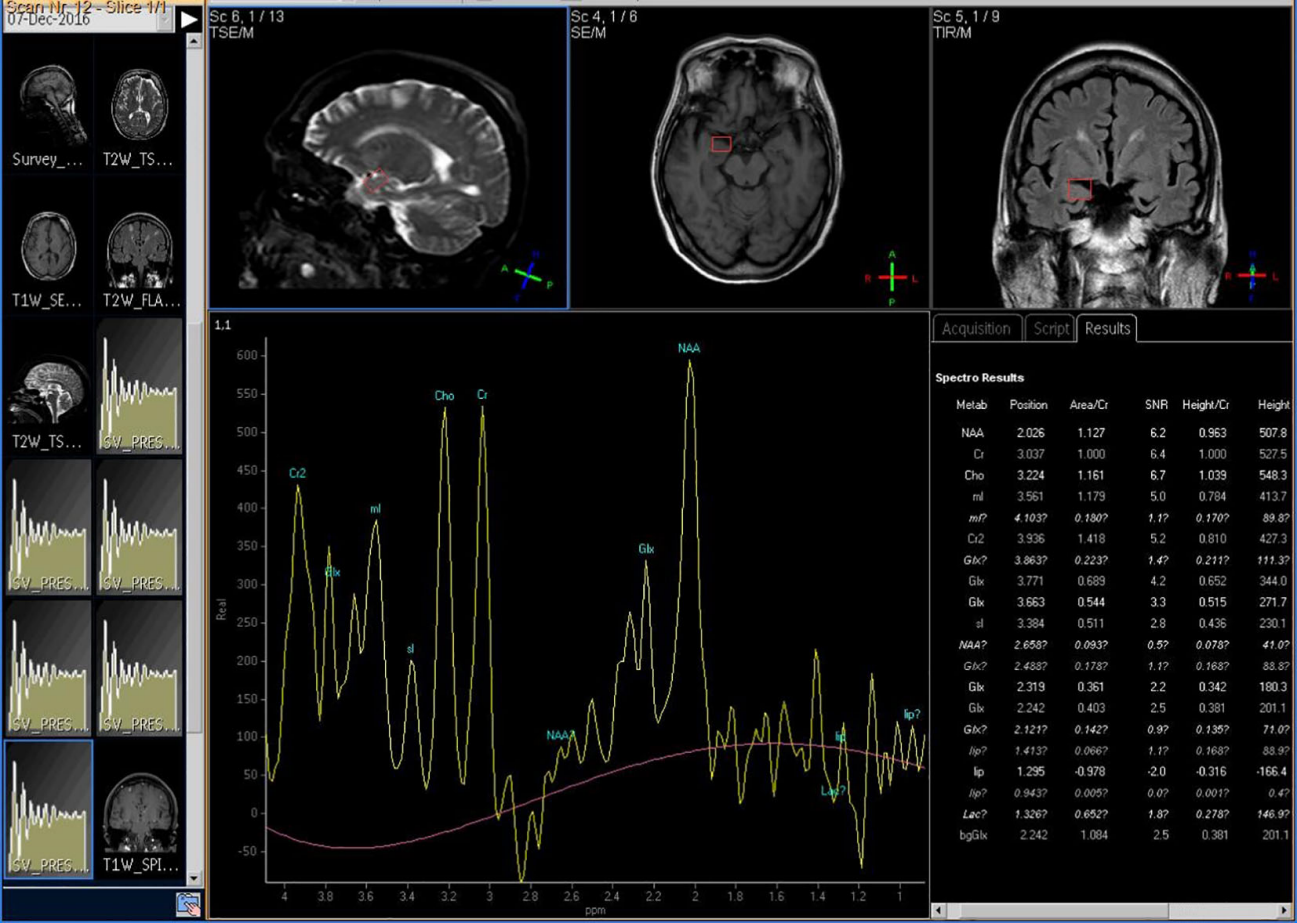
The 1H-MRS imaging of participant's brain metabolism.

The Siemens 3.0T superconducting magnetic resonance imaging system (Magnetom Trio Tim, Siemens, Erlangen, Germany) was used to measure brain metabolic indices. Firstly, routine MRI scanning was performed with SE sequence to exclude brain lesions and locate ^1^H-MRS. Triaxial localization was used in this study. Multi-voxel imaging (MVS) 3D-CSI (chemical shift image) sequence was used for ^1^H-MRS scanning. The repetition time was 1,700 ms and the recovery time was 135 ms. The signal was processed by Philips random software (Achieva 3.0TTx) and converted into data and spectrogram to reflect the relative levels of NAA, Cho, Cr, mI, and Glx (because the absolute quantitative difference of a single metabolite was too large, it was represented by international relative quantitative NAA/Cr, Cho/Cr, mI/Cr, and Glx/Cr). As shown in **Figure 2**, taking Cr as the reference, NAA/Cr, Cho/Cr, mI/Cr, and Glx/Cr were calculated.

### Statistical analysis

Statistical analysis was performed using the IBM SPSS application (version 25.0: IBM, Armonk, NY, USA). Classified data were recorded by absolute frequency and percentage, and quantitative data were recorded by mean and standard deviation.

To assess comparability between the two groups, baseline sociodemographic and clinical characteristics were tested by the *χ*² test and *t*-test. The differences in the outcome variables between the GCMT group and the control group were compared by independent samples *t*-test, and the differences of the outcome variable between baseline and 2 weeks later within the two groups were analyzed using paired samples *t*-test. Pearson correlation was employed to probe the impact factors on the changes in brain metabolic levels. The level of statistical significance was set at *p <*0.05, and the statistical analyses were considered two-tailed.

## Results

### Baseline characteristics of the study groups

A total of 47 participants were included in this study: 31 patients in the GCMTG and 16 patients in the CTRLG. There were 19 men in the GCMTG, with a mean age of 54.87 ± 12.25; 27 were married and 4 separated or widowed; 5 had primary school education, 9 secondary school education, 10 high school or technical school education, and 7 university education or above; 4 were farmers, 9 workers, 13 staff, 2 self-employed individuals, and 3 others; 4 had squamous cell carcinoma, 22 adenocarcinoma, 5 small-cell carcinoma, and 0 adenosquamous carcinoma; 9 were in stage III and 22 in stage IV; 24 patients received chemotherapy, 1 received radiotherapy, and 6 received combined therapy. There were 10 men in the control group, with a mean age of 59.88 ± 8.12; 15 were married and 1 separated or widowed; 3 had primary school education, 4 secondary school education, 7 high school or technical school education, and 2 university education or above; 3 were farmers, 6 workers, 3 staffs, 3 self-employers, and 1 others; 2 had squamous cell carcinoma, 7 adenocarcinoma, 7 small-cell carcinoma, and 0 adenosquamous carcinoma; 5 were in stage III and 11 in stage IV; 8 patients received chemotherapy, 1 received radiotherapy, and 7 received combined therapy. As shown in **Table 1**, there were no significant differences between the two groups on baseline demographics. Within patients with the same cancer stage, there were also no differences between conditions on medical variables.

**Table 1:** Baseline characteristics of the study participants.

Characteristic	GCMTG	CTRLG	*t/χ* ^2^	*p*
Participants (*n*)	31	16		
Mean age (SD)	54.87 ± 12.25	59.88 ± 8.12	2.17	0.15
Sex [*n* (%) men]	19 (61)	10 (63)	0.01	0.94
Marital status [*n* (%)] Married Separated or widowed	27 (87) 4 (13)	15 (94) 1 (6)	0.49	0.48
Education [*n* (%)] Primary school Secondary school High school or technical school University degree or higher	5 (16) 9 (29) 10 (32) 7 (23)	3 (19) 4 (25) 7 (44) 2 (13)	1.05	0.79
Occupation [*n* (%)] Peasant Worker Staff Self-employed Other	4 (13) 9 (29) 13 (42) 2 (6) 3 (10)	3 (19) 6 (38) 3 (19) 3 (19) 1 (6)	3.79	0.44
Pathologic diagnosis [*n* (%)] Squamous cell carcinoma Adenocarcinoma Adenosquamous carcinoma Small-cell carcinoma	4 (13) 22 (71) 0 (0) 5 (16)	2 (13) 7 (44) 0 (0) 7 (44)	4.42	0.11
Stage [*n* (%)] III IV	9 (29) 22 (71)	5 (31) 11 (69)	0.03	0.88
Type of treatment [*n* (%)] Chemotherapy Radiotherapy Combined therapy	24 (77) 1 (3) 6 (19)	8 (50) 1 (6) 7 (44)	3.66	0.16

GCMTG, group computer magnanimous therapy group; CTRLG, control group; SD, standard deviation.

### Magnanimous-enterprising level

After 2 weeks of GCMT, the dimensions of “total score,” “enterprising,” and “magnanimous” (4.87 ± 6.18, 17.06 ± 3.49, and −12.31 ± 4.88) of the GCMTG were higher than baseline (1.77 ± 5.91, 14.94 ± 3.68, and −13.16 ± 4.27). There were significant differences in the dimensions of “total score” and “enterprising” (*p* < 0.05). However, no significant difference was found in the dimension of “magnanimous” (*p* > 0.05).

The “total score,” “enterprising,” and “magnanimous” dimensions in the CTRLG (3.38 ± 5.94, 16.25 ± 2.72, and −12.88 ± 4.52) decreased compared with baseline (4.68 ± 7.68, 17.31 ± 3.63, and −12.63 ± 5.40). There was no significant difference in each of the three dimensions (*p* > 0.05).

Compared with the CTRLG (17.31 ± 3.63), the GCMTG (17.06 ± 3.49) scored significantly lower on the “enterprising” dimension at baseline (*p* < 0.05). However, there were no significant differences between the two groups 2 weeks later. The average difference between baseline and 2 weeks later in the dimension of “enterprising” for the GCMTG was significantly different from the CTRLG (*p* < 0.05). No statistically significant difference between the two groups was evident on the “magnanimous” dimension whether at baseline or after treatment. The difference between baseline and 2 weeks later on “magnanimous” of the two groups was not significant (*p* > 0.05) (**Table 2**).

**Table 2:** Mean values and standard deviations of EMQ at baseline and post-intervention compared with the two groups.

			GCMTG (n = 31)		CTRLG (n = 16)		*t*	*p*
			Mean	SD	Mean	SD		
EMQ	Total score	Baseline	1.77	5.91	4.68	7.68	-1.443	0.156
		2 Weeks later	4.87	6.18	3.38	5.94	0.806	0.430
		*t*	2.529		-1.944		/	/
		*p*	**0.017**		0.071		/	/
		change from baseline	3.10	6.82	-1.31	2.70	2.478	**0.017**
	Enterprising	Baseline	14.94	3.68	17.31	3.63	-2.109	**0.041**
		2 Weeks later	17.06	3.49	16.25	2.72	0.813	0.421
		*t*	2.544		-1.853		/	/
		*p*	**0.016**		0.084		/	/
		change from baseline	2.13	4.66	-1.06	2.29	2.573	**0.013**
	Magnanimous	Baseline	-13.16	4.27	-12.63	5.40	-0.372	0.711
		2 Weeks later	-12.31	4.88	-12.88	4.52	0.464	0.645
		*t*	0.999		-0.845		/	/
		*p*	0.326		0.411		/	/
		change from baseline	0.97	5.39	-0.25 1.18		0.888	0.380

All bold values are significant (*p* < 0.05).

GCMTG, group computer magnanimous therapy group; CTRLG, control group; SD, standard deviation; EMQ, Enterprising and Magnanimous Questionnaire; change from baseline, the mean values of the difference between baseline and 2 weeks later and deviations.

### The evaluation results of brain metabolites

The baseline brain metabolite variables did not have a statistically significant difference between the two groups. The mean level in the NAA/Cr of the right amygdala was significantly increased in the GCMTG, whereas there was no significant difference between the two groups (**Table 3**). The Cho/Cr levels of the left cingulate gyrus and right hippocampus of the control group were significantly higher than 2 weeks ago. However, there were no significant differences between the two groups. The average difference between baseline and 2 weeks later of the mean level of Cho/Cr of the right cingulate gyrus and left amygdala in the intervention group was significantly different from the control group (**Table 4**). The GCMTG had a significantly lower mean level of mI/Cr in the right cingulate gyrus than the CTRLG after 2 weeks. The average difference between baseline and 2 weeks later of the mean level of mI/Cr in the right cingulate gyrus of the GCMTG was clearly distinct from the CTRLG (**Table 5**). There was no significant difference in the ratios of Glx/Cr in the bilateral cingulate gyrus, hippocampus, and amygdala (**Table 6**).

**Table 3:** Mean values and standard deviations of NAA/Cr at baseline and post-intervention compared with the two groups.

			GCMTG (n = 31)		CTRLG (n = 16)		*t*	*p*
			Mean	SD	Mean	SD		
NAA/Cr	L-cingulate gyrus	Baseline	1.71	1.09	1.50	0.29	0.888	0.379
		2 Weeks later	1.54	0.50	1.51	0.23	0.174	0.863
		*t*	0.742		-0.056		/	/
		*p*	0.466		0.956		/	/
		change from baseline	-0.22	1.40	0.00	0.30	-0.559	0.580
	R-cingulate gyrus	Baseline	1.55	0.28	1.53	0.47	0.166	0.869
		2 Weeks later	1.39	0.47	1.51	0.17	-1.009	0.319
		*t*	1.486		0.168		/	/
		*p*	0.150		0.869		/	/
		change from baseline	-0.17	0.61	-0.02	0.51	-0.722	0.475
	L-hippocampus	Baseline	1.36	0.33	1.23	0.50	0.170	0.866
		2 Weeks later	1.56	1.53	1.22	0.50	0.940	0.353
		*t*	-0.667		0.092		/	/
		*p*	0.511		0.928		/	/
		change from baseline	0.20	1.53	-0.01	0.59	0.668	0.509
	R-hippocampus	Baseline	1.51	1.50	1.39	0.51	0.260	0.797
		2 Weeks later	1.32	0.25	1.40	0.29	-0.843	0.404
		*T*	0.609		-0.010		/	/
		*p*	0.548		0.992		/	/
		change from baseline	-0.18	1.54	0.00	0.57	-0.418	0.678
	L-amygdala	Baseline	1.13	0.48	1.19	0.48	0.541	0.593
		2 Weeks later	1.18	0.55	1.11	0.16	0.810	0.425
		*t*	-0.219		0.637		/	/
		*p*	0.833		0.536		/	/
		change from baseline	0.06	0.72	-0.09	0.48	0.539	0.596
	R-amygdala	Baseline	0.67	0.38	1.13	0.39	1.327	0.198
		2 Weeks later	1.13	0.26	1.20	0.32	-0.202	0.842
		*T*	-2.834		-0.603		/	/
		*P*	**0.022**		0.562		/	/
		change from baseline	0.46	0.20	0.07	0.38	-0.596	0.560

All bold values are significant (*p* < 0.05).

NAA, acetylaspartate acid; Cr, creatine; L, left; R, right; GCMTG, group computer magnanimous therapy group; CTRLG, control group; SD, standard deviation; change from baseline, the mean values of the difference between baseline and 2 weeks later and deviations.

**Table 4:** Mean values and standard deviations of Cho/Cr at baseline and post-intervention compared with the two groups.

			GCMTG (n = 31)		CTRLG (n = 16)		*t*	*p*
			Mean	SD	Mean	SD		
Cho/Cr	L-cingulate gyrus	Baseline	1.35	2.02	0.97	0.17	0.722	0.474
		2 Weeks later	1.06	0.59	1.21	0.27	-0.741	0.464
		*t*	0.657		-3.452		/	/
		*p*	0.518		**0.005**		/	/
		change from baseline	-0.30	2.39	0.24	0.25	-0.897	0.376
	R-cingulate gyrus	Baseline	1.18	0.61	1.02	0.34	0.727	0.471
		2 Weeks later	0.93	0.32	1.10	0.24	-1.746	0.089
		*t*	1.991		-0.854		/	/
		*p*	0.058		0.410		/	/
		change from baseline	-0.25	0.62	0.08	0.35	-1.892	**0.046**
	L-hippocampus	Baseline	1.00	0.29	1.15	0.61	-0.972	0.349
		2 Weeks later	1.21	0.74	1.38	0.72	-0.593	0.556
		*t*	-1.953		-1.354		/	/
		*p*	0.062		0.201		/	/
		change from baseline	0.22	0.58	0.23	0.64	-0.168	0.868
	R-hippocampus	Baseline	1.32	1.62	0.86	0.30	0.957	0.345
		2 Weeks later	1.14	0.47	1.20	0.43	-0.628	0.534
		*t*	0.557		-2.341		/	/
		*p*	0.582		**0.037**		/	/
		change from baseline	-0.18	1.66	0.33	0.51	-1.154	0.256
	L-amygdala	Baseline	1.15	0.32	1.03	0.52	1.048	0.305
		2 Weeks later	0.97	0.27	1.22	0.57	-0.519	0.608
		*t*	1.455		-1.818		/	/
		*p*	0.189		0.092		/	/
		change from baseline	-0.18	0.35	0.18	0.27	-3.362	**0.003**
	R-amygdala	Baseline	1.24	0.50	1.10	0.36	1.439	0.164
		2 Weeks later	1.27	0.44	1.47	0.45	-0.570	0.574
		*t*	-0.200		-2.081		/	/
		*p*	0.848		0.067		/	/
		change from baseline	0.03	0.29	0.37	0.56	-1.495	0.156

All bold values are significant (*p* < 0.05).

Cho, choline; Cr, creatine; L, left; R, right; GCMTG, group computer magnanimous therapy group; CTRLG, control group; SD, standard deviation; change from baseline, the mean values of the difference between baseline and 2 weeks later and deviations.

**Table 5:** Mean values and standard deviations of mI/Cr at baseline and post-intervention compared with the two groups.

			GCMTG (n = 31)		CTRLG (n = 16)		*t*	*p*
			Mean	SD	Mean	SD		
mI/Cr	L-cingulate gyrus	Baseline	0.76	0.63	0.71	0.24	0.223	0.825
		2 Weeks later	0.60	0.54	0.75	0.19	-0.634	0.532
		*t*	0.208		-0.311		/	/
		*p*	0.838		0.766		/	/
		change from baseline	-0.16	0.73	0.04	0.34	-0.424	0.676
	R-cingulate gyrus	Baseline	0.76	0.64	0.73	0.20	-0.437	0.665
		2 Weeks later	0.42	0.20	0.75	0.16	-3.494	**0.002**
		*t*	2.167		-0.821		/	/
		*p*	**0.046**		0.443		/	/
		change from baseline	-0.33	0.23	0.03	0.08	-3.134	**0.005**
	L-hippocampus	Baseline	0.67	0.41	0.79	0.17	-0.440	0.663
		2 Weeks later	0.58	0.19	0.80	0.11	-1.493	0.147
		*t*	0.860		-0.020		/	/
		*p*	0.402		0.985		/	/
		change from baseline	-0.11	0.34	0.00	0.20	-0.763	0.454
	R-hippocampus	Baseline	1.10	1.83	0.63	0.28	0.723	0.475
		2 Weeks later	0.67	0.21	0.80	0.26	-1.931	0.064
		*t*	0.963		-1.213		/	/
		*p*	0.349		0.279		/	/
		change from baseline	-0.44	1.93	0.17	0.34	-0.757	0.457
	L-amygdala	Baseline	0.82	0.73	0.80	0.33	0.100	0.922
		2 Weeks later	0.65	0.18	0.93	0.25	-2.147	**0.047**
		*t*	-0.387		-1.439		/	/
		*p*	0.712		0.193		/	/
		change from baseline	-0.16	0.32	0.14	0.27	-0.581	0.571
	R-amygdala	Baseline	0.93	0.19	0.87	0.38	0.648	0.524
		2 Weeks later	0.83	0.30	0.80	0.23	1.438	0.172
		*t*	0.749		0.502		/	/
		*p*	0.488		0.633		/	/
		change from baseline	-0.10	0.29	-0.06	0.59	-0.482	0.641

All bold values are significant (*p* < 0.05).

mI, myo-inositol; Cr, creatine; L, left; R, right; GCMTG, group computer magnanimous therapy group; CTRLG, control group; SD, standard deviation; change from baseline, the mean values of the difference between baseline and 2 weeks later and deviations.

**Table 6:** Mean values and standard deviations of Glx/Cr at baseline and post-intervention compared with the two groups.

			GCMTG (n = 31)		CTRLG (n = 16)		*t*	*p*
			Mean	SD	Mean	SD		
Glx/Cr	L-cingulate gyrus	Baseline	0.63	0.50	0.41	0.21	1.615	0.116
		2 Weeks later	0.62	0.49	0.32	0.05	1.640	0.115
		*t*	0.057		0.996		/	/
		*p*	0.955		0.358		/	/
		change from baseline	0.02	0.66	-0.09	0.25	0.445	0.661
	R-cingulate gyrus	Baseline	0.45	0.23	0.52	0.15	0.643	0.525
		2 Weeks later	0.51	0.29	0.43	0.12	1.830	0.080
		*t*	-1.034		1.334		/	/
		*p*	0.317		0.231		/	/
		change from baseline	0.05	0.22	-0.09	0.27	0.711	0.482
	L-hippocampus	Baseline	0.72	0.25	0.53	0.15	1.709	0.090
		2 Weeks later	0.65	0.28	0.50	0.17	1.293	0.208
		*t*	0.651		0.945		/	/
		*p*	0.525		0.388		/	/
		change from baseline	-0.06	0.40	-0.03	0.17	-0.192	0.850
	R-hippocampus	Baseline	0.72	0.43	0.40	0.18	1.206	0.237
		2 Weeks later	0.67	0.51	0.53	0.19	0.652	0.520
		*t*	0.373		-1.745		/	/
		*p*	0.714		0.141		/	/
		change from baseline	-0.05	0.58	0.13	0.18	-0.755	0.459
	L-amygdala	Baseline	0.88	0.93	0.57	0.35	1.622	0.131
		2 Weeks later	0.76	0.68	1.02	0.90	-0.753	0.463
		*t*	0.243		-1.240		/	/
		*p*	0.816		0.255		/	/
		change from baseline	-0.12	1.27	0.45	1.03	-0.954	0.357
	R-amygdala	Baseline	0.62	0.13	0.57	0.23	1.655	0.113
		2 Weeks later	0.44	0.19	0.65	0.21	-0.631	0.538
		*t*	2.521		-1.064		/	/
		*p*	0.053		0.328		/	/
		change from baseline	-0.20	0.46	0.08	0.18	0.443	0.667

Glx, glutamate and glutamine complex; Cr, creatine; L, left; R, right; GCMTG, group computer magnanimous therapy group; CTRLG, control group; SD, standard deviation; change from baseline, the mean values of the difference between baseline and 2 weeks later and deviations.

### The relationship between the changes of magnanimous-enterprising level and brain biochemical metabolism

**Figure 3:**
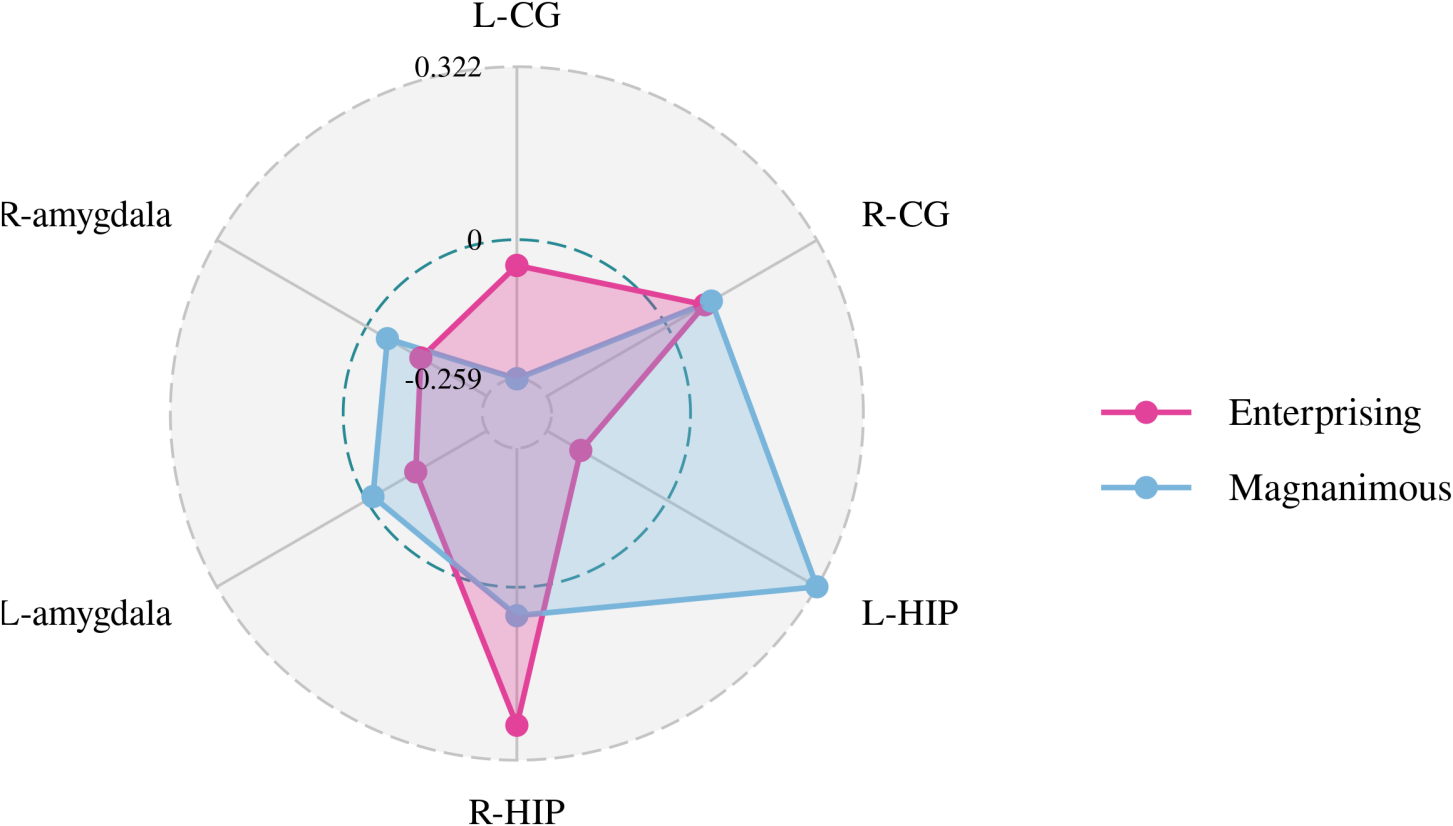
Pearson correlation radar chart of changes in magnanimous-enterprising level and NAA/Cr metabolism in various brain regions.

**Figure 4:**
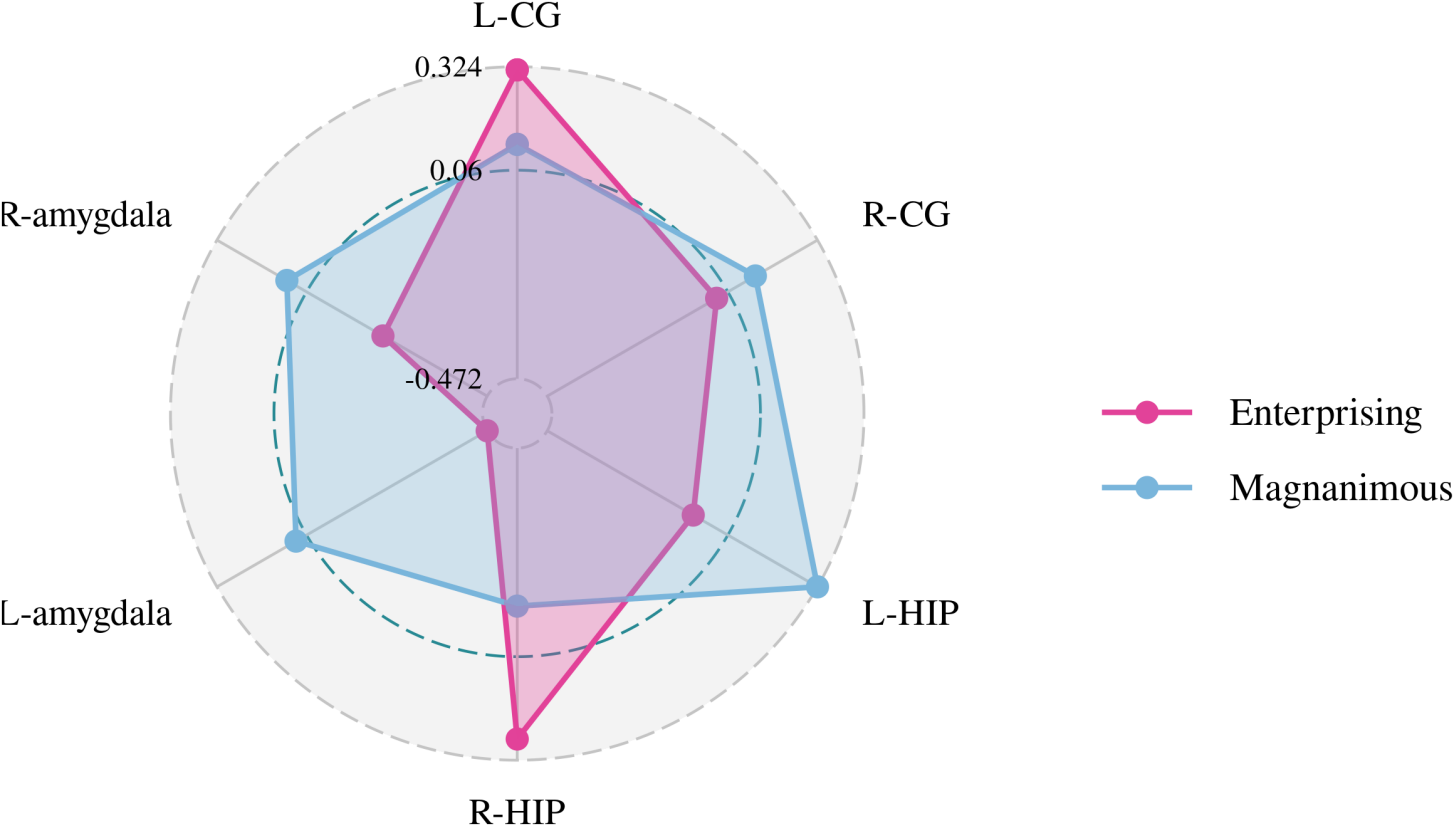
Pearson correlation radar chart of changes in magnanimous-enterprising level and Cho/Cr metabolism in various brain regions.

**Figure 5:**
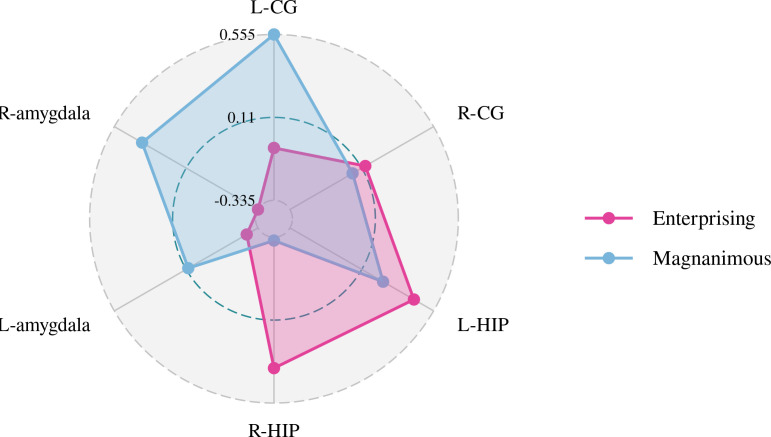
Pearson correlation radar chart of changes in magnanimous-enterprising level and Glx/Cr metabolism in various brain regions.

**Figure 6:**
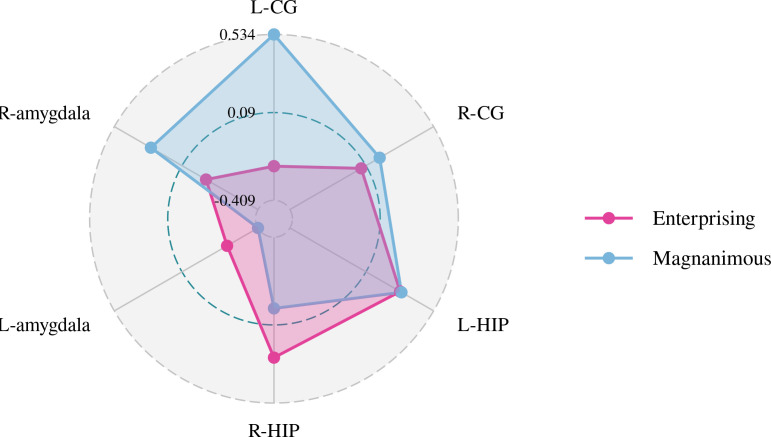
Pearson correlation radar chart of changes in magnanimous-enterprising level and mI/Cr metabolism in various brain regions.

A Pearson correlation analysis of the changes from baseline between the biochemical metabolism of the brain regions and EMQ scores was conducted to investigate the possible neuroanatomical mechanism. The results showed that changes in the mean level of Cho/Cr in the left amygdala and Glx/Cr in the left hippocampus were significantly correlated to the changes in enterprising level (**Figures 3**–**5**). The changes in individual magnanimous level had a significant correlation with the changes of mI/Cr level and Glx/Cr in the left cingulate gyrus (**Figures 3**, **5**, **6**).

## Discussion

In China, previous studies have reported that per capita medical resources are relatively scarce, and the population’s health awareness of cancer prevention is comparatively poor. A total of 75% of lung cancer patients were diagnosed at the advanced stage and missed the best treatment period (30). Although tumor diagnosis and treatment technology have made great progress, we are still helpless in the face of the most advanced tumors. Therefore, it is particularly important to use psychotherapy to intervene with the psychosomatic status of cancer patients, assist in clinical treatment, and improve the long-term quality of life of cancer patients.

As a new psychotherapy for patients with advanced lung cancer, GCMT significantly improved the levels of enterprising and brain metabolism in our study. After the intervention, the “total score” and “enterprising” dimensions of EMQ revealed a significant improvement in the GCMTG, and the magnitude of the changes from baseline was significantly different from the control group. After 2 weeks, NAA/Cr of the right amygdala and mI/Cr of the right cingulate gyrus showed significant (*p* < 0.05) changes in the GCMTG. Although there was no difference between baseline and 2 weeks later of Cho/Cr in the left amygdala and right cingulate gyrus in either the GCMTG or the CTRLG, there was a statistically significant difference when comparing the changes over 2 weeks between the two groups.

It has been found that the mental health status of cancer patients corresponds to their personality traits (introversion, loneliness, stubbornness, emotional imbalance, obsessive–compulsive symptoms and thinking, maladjustment, interpersonal sensitivity) (31, 32). At the same time, studies have shown that the occurrence, development, and prognosis of tumors are closely related to the psychological personality factors of patients (33). Cancer patients with long-term survival and quality of life generally have common psychological characteristics of positive optimism, balanced understanding, open-minded tolerance, and good fortune (34). Therefore, a positive and open-minded attitude can delay the occurrence and development of cancer and affect its prognosis, which is the key factor to improve the quality of life and extend the lifespan of cancer patients (35–37). Our study showed that GCMT was helpful in cultivating a positive and enterprising belief in hospitalized patients with lung cancer, which showed that GCMT has a positive significance for clinical rehabilitation and long-term survival of patients with advanced lung cancer.

In the treatment stage, through the guidance and inspiration of the therapist, the patients experienced gradual realization or epiphany; through interaction and communication among group members, GCMT aroused resonance and reflection, promoted the growth of members, deepened their understanding of life, improved the curative effect, enabled patients to cooperate with the clinical treatment of lung cancer with a positive attitude, established effective social support, and helped patients reduce loneliness caused by cancer; these are made possible through repeated learning, remembering, and perceiving the enterprising and magnanimous information and applying to solve the problems in daily life, leading to an enterprising and magnanimous state of mind. Through mutual encouragement among group members and the sharing of successful cases, the levels of the “enterprising” dimension have changed significantly.

Aside from the observation of the psychological advantages of GCMT, we were especially encouraged by the observed improvement in brain metabolism. Although no significant changes in brain metabolism were observed in most brain regions in the GCMTG before and after the intervention, which was consistent with the research results of Yang R (38) and Bao H (39), the results suggest that the NAA/Cr level in the right amygdala in the intervention group increased significantly, while the NAA/Cr level in the left hippocampus and left amygdala tended to increase, which was similar to other studies (40). NAA widely exists in the neurons and is responsible for the integrity of neurons. Many studies have shown that the NAA/Cr ratio of the prefrontal cortex and anterior cingulate cortex was significantly associated with neuronal damage, cognitive decline, and unhealthy emotions (41–43). Therefore, it can be inferred that GCMT can improve patients’ negative emotions and functional abilities, which also confirms the results of our previous psychological measurements (27, 28) from the perspective of neuroimaging.

There was a significant difference in Cho/Cr in the right cingulate gyrus and left amygdala between the GCMTG and the CTRLG before and after treatment (*p* < 0.05). The Pearson correlation analysis of the changes from baseline and 2 weeks later of the indicators for the study groups showed that the changes of Cho/Cr in the left amygdala and Glx/Cr in the left hippocampus may be the factors that drive the improvements of enterprising mentality. Therefore, it can be inferred that the impact of GCMT on enterprising attitude may be related to the reduction of Cho/Cr in the left amygdala. Studies showed that the left amygdala may be related to negative emotions such as anger, fear, sadness, and continuous daily painful emotional experiences (44, 45). It was speculated that the decreased level of Cho/Cr in the amygdala leads to improvement of negative emotional experiences. In other words, GCMT was probable to make patients more confident in the treatment and lead to positive changes by improving patients’ daily negative emotional experiences. A meta-analysis of proton magnetic resonance (^1^H-MRS) spectroscopy studies (11) showed that reduced levels of Glx metabolites in the medial prefrontal cortex may be associated with the pathophysiology of depression (SMD = −0.38; 95% CI, −0.69 to −0.07). However, due to technical localization reasons, we were unable to measure the Glx/Cr in the medial prefrontal cortex of lung cancer patients. We will supplement the brain metabolism studies of the medial prefrontal cortex in subsequent experiments and further explore the intrinsic relationship between emotions such as depression or anxiety and magnanimous-enterprising levels.

Based on Moher D and Newell’s recommendations and revised CONSORT statement (46), we clearly and in detail explained the procedure of this study in the “study design” section, taking full account of the sociodemographic and clinical characteristics of all patients to control bias. In addition, each volunteer was followed up. The manner and process of intervention have been described above. The study design has been examined by oncologists, statisticians, other psychologists, and psychiatrists, which was considered scientific and strict.

There are several limitations in the present study. First, we failed to observe significant changes in the “magnanimous” dimension, which was probably due to the limitation of intervention time and the stability of psychological and behavioral habits. Further research is warranted to assess the long-term effects. Secondly, our strict inclusion and exclusion criteria lowered our sample size, which made the statistical results prone to deviation. Further research with larger sample sizes is needed to confirm our results. Third, with the limitation of experimental conditions, we were unable to collect data from other brain regions like the striatum and thalamus, which are closely related to the generation and regulation of emotion. Further studies to investigate the mechanism of the improvements of cerebral metabolic functions for GCMT are needed.

## Conclusion

We demonstrated that GCMT has positive short-term effects on the enterprising levels and brain metabolic functions of patients with advanced lung cancer. Patients who received GCMT intervention exhibited a significant increase in their scores on the “enterprising” dimension and a notable decrease in the Cho/Cr ratio in the left amygdala. This suggests that GCMT may improve patients’ psychological states by influencing the metabolic activity of emotion-related brain regions.

These results provide empirical support for the application of GCMT in patients with advanced lung cancer, expanding the potential of psychological interventions in cancer treatment. Future studies should further validate these findings and explore the long-term effects and underlying mechanisms of GCMT.

## Data Availability

The original contributions presented in the study are included in the article/**Supplementary Material**. Further inquiries can be directed to the corresponding author.
