# Supplementary material for: The cerebral metabolic mechanism of group computer magnanimous therapy based on magnetic resonance spectroscopy: effects on improving magnanimous-enterprising levels of lung cancer patients
Source: Front Psychiatry. 2024 Dec 10;15:1397375. doi: 10.3389/fpsyt.2024.1397375 (PMC11666494; doi:10.3389/fpsyt.2024.1397375)
Supplement: Supplementary file 2 [file Table2.docx]

Supplementary table

**TABLE7-1** Pearson correlation between the changes of magnanimous-enterprising level and brain biochemical metabolism

| NAA/Cr | | | | | L-CG | | R-CG | | L-HIP | R-HIP | L-amygdala | R-amygdala |  |
| --- | --- | --- | --- | --- | --- | --- | --- | --- | --- | --- | --- | --- | --- |
| EMQ | | | Enterprising | | Pearson correlation | | -0.048 | | 0.081 | -0.186 | 0.257 | -0.106 | -0.117 |
|  |  | | | *p* | | 0.786 | | 0.628 | 0.271 | 0.114 | 0.647 | 0.653 |  |
|  | Magnanimous | | | Pearson correlation | | -0.259 | | 0.095 | 0.322 | 0.053 | -0.014 | -0.045 |  |
|  |  | | *p* | | | 0.132 | | 0.569 | 0.052 | 0.750 | 0.951 | 0.863 |  |

NAA: acetylaspartic acid; Cr: creatine; CG: cingulate gyrus; HIP: hippocampus; L: left; R: right; EMQ: Enterprising and Magnanimous Questionnaire; All bold values are significant (*p* < 0.05).

**TABLE7-2** Pearson correlation between the changes of magnanimous-enterprising level and brain biochemical metabolism

| Cho/Cr | | | | L-CG | | R-CG | | L-HIP | R-HIP | L-amygdala | | R-amygdala |  |
| --- | --- | --- | --- | --- | --- | --- | --- | --- | --- | --- | --- | --- | --- |
| EMQ | | | Enterprising | Pearson correlation | | 0.316 | | 0.027 | -0.042 | 0.270 | | -0.472 | -0.165 |
|  |  | | *p* | | 0.060 | | 0.871 | 0.803 | 0.097 | | **0.031** | 0.528 |  |
|  | Magnanimous | | Pearson correlation | | 0.126 | | 0.141 | 0.324 | -0.069 | | 0.091 | 0.118 |  |
|  |  | | *p* | | 0.463 | | 0.397 | 0.050 | 0.677 | | 0.695 | 0.651 |  |

Cho: choline; Cr: creatine; CG: cingulate gyrus; HIP: hippocampus; L: left; R: right; EMQ: Enterprising and Magnanimous Questionnaire; All bold values are significant (*p* < 0.05).

**TABLE7-3** Pearson correlation between the changes of magnanimous-enterprising level and brain biochemical metabolism

| mI/Cr | | | | L-CG | R-CG | L-HIP | R-HIP | L-amygdala | | R-amygdala |  |
| --- | --- | --- | --- | --- | --- | --- | --- | --- | --- | --- | --- |
| EMQ | | | Enterprising | Pearson correlation | -0.215 | 0.059 | 0.314 | 0.276 | | -0.206 | -0.068 |
|  |  | | *p* | 0.349 | 0.789 | 0.144 | 0.192 | | 0.461 | 0.852 |  |
|  | Magnanimous | | Pearson correlation | 0.534 | 0.180 | 0.323 | -0.004 | | -0.409 | 0.294 |  |
|  |  | | *p* | **0.013** | 0.412 | 0.133 | 0.986 | | 0.130 | 0.410 |  |

mI: myo-inositol; Cr: creatine; CG: cingulate gyrus; HIP: hippocampus; L: left; R: right; EMQ: Enterprising and Magnanimous Questionnaire; All bold values are significant (*p* < 0.05).

**TABLE7-4** Pearson correlation between the changes of magnanimous-enterprising level and brain biochemical metabolism

| Glx/Cr | | | | L-CG | R-CG | L-HIP | R-HIP | L-amygdala | R-amygdala | |
| --- | --- | --- | --- | --- | --- | --- | --- | --- | --- | --- |
| EMQ | | | Enterprising | Pearson correlation | -0.054 | 0.132 | 0.433 | 0.368 | -0.265 | -0.335 |
|  |  | | *p* | 0.813 | 0.559 | **0.044** | 0.084 | 0.339 | 0.344 |  |
|  | Magnanimous | | Pearson correlation | 0.555 | 0.053 | 0.242 | -0.317 | 0.097 | 0.383 |  |
|  |  | | *p* | **0.007** | 0.816 | 0.278 | 0.141 | 0.730 | 0.274 |  |

Glx: glutamate and glutamine complex; Cr: creatine; CG: cingulate gyrus; HIP: hippocampus; L: left; R: right; EMQ: Enterprising and Magnanimous Questionnaire; All bold values are significant (*p* < 0.05).
